# Factors associated with seedling establishment on logs of different fungal decay types—A seed‐sowing experiment

**DOI:** 10.1002/ece3.11508

**Published:** 2024-06-04

**Authors:** Yu Fukasawa, Hiroyuki Kitabatake

**Affiliations:** ^1^ Laboratory of Forest Ecology, Graduate School of Agricultural Science Tohoku University Osaki Japan

**Keywords:** coarse woody debris, microsites for tree regeneration, mycorrhizal type, *Pinus densiflora*, plant–soil feedback, rot type, wood‐inhabiting fungi

## Abstract

Wood decay fungi alter the abiotic and biotic properties of deadwood, which are important as nurse logs for seedling regeneration. However, the relationship between fungal decay type and seedling performance has not been evaluated experimentally. In this study, we examined the germination, growth, and survival of six arbuscular mycorrhizal (AM) and six ectomycorrhizal (ECM) tree species on three substrates (pine logs with brown and white rot and soil) by conducting seed‐sowing experiments in a mixed forest dominated by *Pinus densiflora* and *Quercus serrata*. Analysis using ribosomal DNA internal transcribed spacer 1 (rDNA ITS1) sequencing revealed that the fungal community was significantly different across three substrates. The richness of operational taxonomic units (OTUs) of AM and ECM fungi was the largest on brown rot logs and soil, respectively. The substrate significantly affected the seedling performance when comparing wood decay types, but these were not consistent across the mycorrhizal status of the seedlings. Nevertheless, seedlings of some AM trees showed better growth and enhanced mycorrhizal colonization on brown rot logs than on white rot logs. The wood decay type influenced fungal communities in the logs and the performance of some seedling species partly by different mycorrhizal colonization rates. However, the effect was seedling species dependent and showed no apparent difference between AM and ECM trees.

## Graphical abstract

We examined germination, growth, and survival of six arbuscular mycorrhizal and six ectomycorrhizal tree species on three substrates (brown rot logs, white rot logs, and soil) by seed sowing experiments. The decay type (brown rot and white rot) affected seedling performance, depending on the seedling species, and the difference between the two mycorrhizal types was not clear. The effects of fungal wood decay type on nurse log regeneration of tree seedlings might be partly attributable to the difference in microbial communities within logs.

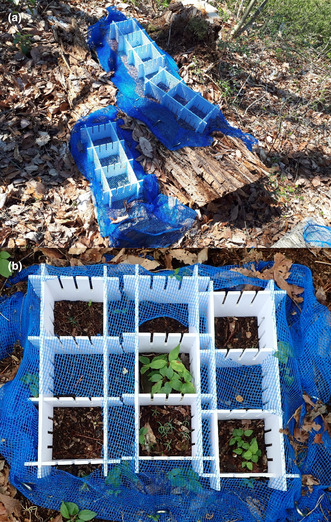

## 1 INTRODUCTION

Nurse logs are vital for the regeneration of forest trees, not only in boreal and subalpine coniferous forests but also in temperate and tropical broad‐leaved forests (Christie & Armesto, 2003; Doi et al., 2008; Harmon & Franklin, 1989; Papaik & Canham, 2006; Sanchez et al., 2009). Decaying logs provide regeneration microsites with good light conditions, reduced litter accumulation, low root competition, and abundant water content. These conditions are particularly advantageous for small‐seeded species with limited energy reserves for initial growth, which can still become canopy dominants (Lusk, 1995). Understanding the mechanisms and factors associated with tree regeneration on nurse logs is crucial for predicting forest dynamics.

The properties of the logs affecting seedling regeneration have been extensively studied. For instance, thick logs host many seedlings (Takahashi, 1994). Well‐decayed logs that are softened, moistened, and covered with abundant moss provide better microsites for seedlings compared to undecayed, hard logs without a moss layer (Fukasawa & Ando, 2018; Iijima & Shibuya, 2010; Mori et al., 2004). Additionally, the tree species of the logs must be considered (Orman et al., 2016). The decay type of logs, including the physicochemical properties of decaying wood caused by fungal decomposers, has recently been considered an important factor influencing seedling regeneration (Fukasawa, 2021). The decay type is fungal species‐ or strain‐specific preferences for different components of wood, such as lignin, cellulose, and hemicellulose (Fukasawa, 2021). The decay type of basidiomycetes, a phylogenetic group of fungi with strong wood decay abilities, traditionally falls into two categories: brown rot and white rot (Eaton & Hale, 1993). In white rot, lignin is selectively or simultaneously decayed along with cellulose and hemicellulose, resulting in fibrous, soft, spongy wood due to the decomposition of the lignin binding the cell walls (Araya, 1993). Contrastingly, brown rot mainly targets cellulose and hemicellulose, leaving lignin with little modification. Wood affected with brown rot turns brown and has a blocky texture. As brown rot formation requires acidic conditions (Espejo & Agosin, 1991), wood affected with brown rot wood is more acidic than those with white rot (Fukasawa, 2012). Differences in wood decay type can significantly affect seedling communities on the logs, potentially leading to niche separation among dominant tree seedlings in a local context (Fukasawa et al., 2017). However, the factors determining the effect of decay types on tree seedling regeneration are poorly understood.

Previous field studies have reported that tree seedlings mainly associated with arbuscular mycorrhizal (AM) fungi tend to regenerate more frequently on brown rot logs than on white rot logs (Fukasawa, 2021). In contrast, seedlings mainly associated with ectomycorrhizal (ECM) fungi tend to regenerate more frequently on white rot logs than on brown rot logs (Fukasawa, 2021). These mycorrhizal types are formed by distinct fungal groups and are related to different phylogenetic groups of trees (Smith & Read, 2008). Mycorrhizal fungi are abundant in decayed wood in forests, especially in the later decay stages (Rajala et al., 2011, 2012, 2015). They are essential for seedling colonization on the logs (Fukasawa, 2012; Marx & Wakters, 2006). Tedersoo et al. (2008) reported that the wood decay type influences the ECM fungal communities associated with the roots of tree seedlings growing on the logs. Therefore, difference in mycorrhizal fungi on decaying logs might explain the effect of the wood decay type on seedling regeneration. However, the communities of AM fungi in white and brown rot logs and the seedling performance on decaying logs with different decay types have not been compared among seedlings of different mycorrhizal types.

In the present study, we evaluated the effects of abiotic and biotic properties of three substrates, including brown and white rot logs and soil, on tree seedling establishment in a secondary mixed forest. We achieved this by sowing the seeds of six AM and six ECM tree species in the field. The fungal communities in the substrates were analyzed using metabarcoding of fungal DNA directly extracted from the samples of each substrate. We hypothesized that the three substrates have distinct fungal communities. Particularly, ECM fungal species, which can decay organic matter, might be more predominant in white rot logs than brown rot logs due to their high‐cellulose and hemicellulose content. Conversely, AM fungi, which generally cannot decay organic matter, were predicted to occur more frequently on brown rot logs. The high concentration of recalcitrant lignin in brown rot logs might suppress colonization by saprotrophic fungi (Lindner et al., 2011), providing AM fungi a competitive advantage on the roots of host plants. Seedling establishment, particularly in its early stages, is determined by three key processes: germination, survival, and growth. We hypothesized that the seedlings of AM trees might exhibit better growth and survival on brown rot logs than on white rot logs or soil. In contrast, the seedlings of ECM trees might perform better on white rot logs than on brown rot logs or in soil due to the relative dominance of their mycorrhizal symbionts.

## 2 MATERIALS AND METHODS

### 2.1 Study site

This study was conducted in a secondary forest dominated by oak (*Quercus serrata*), pine (*Pinus densiflora*), and cedar (*Cryptomeria japonica*) in Mt. Chitose (38°14′ N 140°21′ E, altitude: 245 m) located in the northern part of the central island of Japan. The site features a gentle northwestern slope with a mean total annual precipitation of 1207 mm, a mean annual temperature of 12.1°C (for the period 1991–2020, according to the Japan Meteorological Agency), and a maximum snow depth of approximately 1 m at the nearest weather station (Yamagata; 38°15′ N 140°21′ E, altitude: 153 m). This area is a shrine forest managed by the Forestry Agency of Japan.

Pine wilt disease, caused by the North American native pinewood nematode *Bursaphelenchus xylophilus*, was first observed in this area in 1982, causing significant destruction of *P. densiflora* over the past few decades. To prevent the spread of this disease, the region has undergone deadwood management, including felling infected trees and fumigating with pesticides, such as methylcarbamodithioic acid ammonium (NCS). Notably, this forest floor lacks dwarf bamboo *Sasa* spp., which typically dominates the understory of many Japanese forests. Previous studies conducted on this site have reported that seedlings of *C. japonica*, *P. densiflora*, *Clethra barbinervis*, and *Ilex crenata* were frequently observed on *P. densiflora* logs (Fukasawa et al., 2017). Moreover, the growth and survival of *C. japonica* seedlings were shown to be negatively correlated with white rot logs compared with brown rot logs (Fukasawa & Komagata, 2017).

### 2.2 Seed‐sowing experiment

**FIGURE 1:**
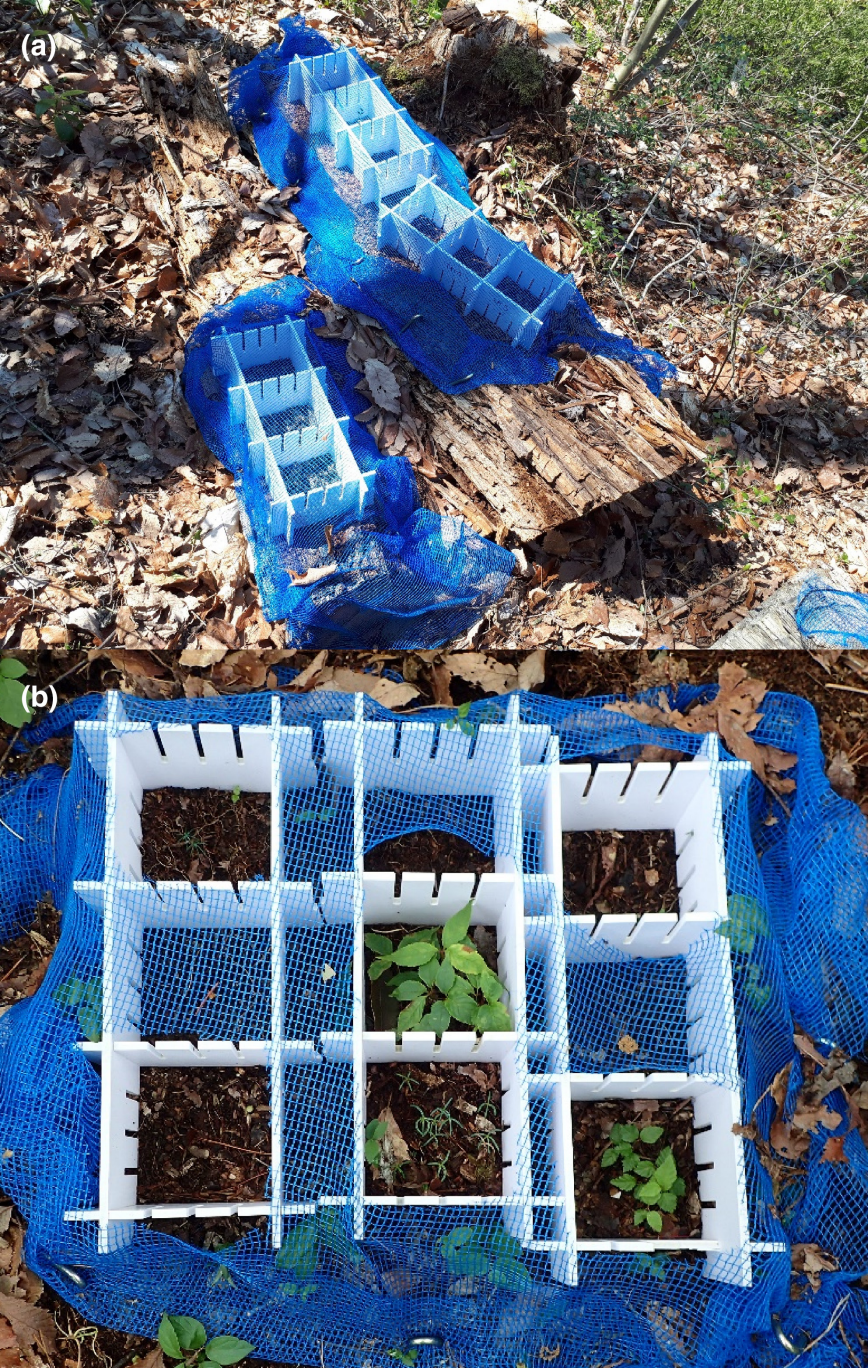
Arrangements of 5 × 5 cm quadrats on logs (a) and soil (b). The mesh cover on each quadrat was removed after germination.

In an approximately 1 ha tract at the study site, we selected 62 *P. densiflora* logs (diameter 19–62, length 62–200 cm) at 10 locations. These logs were well‐decayed, falling into decay class IV within a five‐class decay system (Fukasawa, 2012). Furthermore, 30 white and 32 brown rot logs were identified based on visual criteria (Araya, 1993). To prepare the logs, we removed the moss layer and surface vegetation from all the logs (we did not record their species) and plowed and flattened the tops using a hand axe. The interior of some of the logs had plant roots growing from the soil and other remains of surface vegetation. We kept these roots as it was impossible to remove all of them. We set up multiple quadrats (5 × 5 cm) on the logs by surrounding them with polyvinyl chloride plates (9 mm height with slits) (AooYoo, ShenZhen, China) (Figure 1). Similar quadrats were established on the ground soil at the exact 10 locations after removing the litter layer.

Mature seeds of six AM and six ECM tree species were collected from the study site or nearby regions (Table 1). The seeds for *Abies veitchii*, *Betula ermanii*, *Chamaecyparis obtusa*, and *Cryptomeria japonica* were obtained from the public seed collection of the Forestry and Forest Products Research Institute (Ibaraki, Japan). *Picea jezoensis* seeds were sourced from a private company (H.I.Tree C's, Saitama, Japan) because it was hard to collect sufficient seeds due to a non‐masting season in the study area. The seeds were sown in November 2019 (for 21 white rot and 23 brown rot logs) and November 2020 (for additional logs of 9 white rot and 9 brown rot). We prepared nine replicate quadrats for each species on the three substrates (brown and white rot log and soil). Seed size and predicted germination rate of each species were considered when deciding seed number sown in a quadrat (35–120 seeds/quadrat, Table 1) to prevent seedlings from becoming too dense after germination. In total, 19,170 seeds were sown. To protect the seeds from wind and small mammals during the winter, the quadrats were covered with 4 mm mesh nets and securely fastened with metal pegs onto the substrates (Figure 1).

**TABLE 1:** List of seed species used, their mycorrhizal types, and the number of seeds sown in each quadrat.

Species	Mycorrhizal type	Seed weight (g)^a^	Sowing	Number per quadrat
*Chamaecyparis obtusa* (Siebold et Zucc.) Endl.	AM	3.19	Nov. 2020	70
*Clethra barbinervis* Siebold et Zucc.	AM	0.49	Nov. 2019	120
*Cryptomeria japonica* (L.f.) D. Don	AM	4.07	Nov. 2019	40
*Ilex crenata* Thunb. var. *crenata*	AM	12.00	Nov. 2020	35
*Padus grayana* (Maxim.) C.K. Schneid.	AM	59.90	Nov. 2019	60
*Toxicodendron trichocarpum* (Miq.) Kuntze	AM	20.00	Nov. 2020	40
*Abies veitchii* Lindl.	ECM	8.94	Nov. 2019	60
*Alnus hirsuta* Turcz. var. *sibirica (*Spach) C.K. Schneid.	ECM	0.68	Nov. 2020	60
*Betula ermanii* Cham.	ECM	0.62	Nov. 2020	50
*Carpinus laxiflora* (Siebold et Zucc.) Blume	ECM	3.00	Nov. 2019	60
*Picea jezoensis* (Siebold et Zucc.) Carrière var. *jesoensis*	ECM	2.42	Nov. 2019	55
*Pinus densiflora* Siebold et Zucc.	ECM	9.21	Nov. 2019	60

^a^ Air‐dried weight of 1000 seeds (Seed Information Database (https://ser‐sid.org, accessed May 2, 2024); Osada (2005) for *T. trichocarpum* = *Rhus trichocarpa*).

Starting the following spring (either April 2020 or April 2021), we recorded seed germination and survival for two growing seasons for seeds sown in 2019 and one growing season for seeds sown in 2020 until October 2021. We recorded the number of live seedlings in each quadrat without individual tagging. These recordings were conducted 13 times in 2020 (from April to November) and 10 times in 2021 (from April to October), including observations every 2 weeks from April to August and monthly thereafter.

In October 2021, all the seedlings were harvested and transported to the laboratory. Fresh shoot lengths were measured, and the dried weights of shoots and roots were recorded after drying for over 3 days at 70°C. Fresh root sub‐samples were obtained from several seedlings to determine the colonization rate of mycorrhizal fungi.

### 2.3 Physicochemical properties of logs and soil

The canopy openness above each log and soil quadrat was documented by capturing hemispherical images on a cloudy day in June 2020 (for 44 logs and 10 soil subplots) and June 2021 (for additional 18 logs) using a Canon EOS Kiss X5 camera equipped with a circular fisheye lens (4.5 mm F2.8 EX DC, SIGMA, Kanagawa, Japan). To compute the canopy openness, we employed the CanopOn 2 software (available at http://takenaka‐akio.org/etc/canopon2/). The water content of the logs and soil subplots was also measured using a portable soil moisture meter DIK‐31F (Daiki, Saitama, Japan) during each seedling survey. The time‐series data for water content were averaged for each substrate and subsequently used for the following analyses.

In September 2020 (for 44 logs and 10 soil quadrats) and September 2021 (for additional 18 logs), substrate samples were obtained manually using rubber gloves or knives due to the softness of the log surfaces. From each substrate (individual logs and soil quadrat), three samples were collected and then combined into a single sample (a total of 72 combined samples), approximately 100 mL each. These samples were transported back to the laboratory and kept in a fridge at around 8°C for a week until chemical analysis.

The samples were pulverized using a blender WB‐1 (Osaka Chemical, Osaka, Japan) and passed through a 5 mm mesh. Crushed samples (ca. 60 mL) were subjected to extraction with 200 mL of deionized water in 250 mL polyethylene bottles for 1 h of shaking (100 rpm on a Shaker MK201D) (Yamato Scientific, Tokyo, Japan). The pH of the extract was measured using a portable pH meter (LAQUAtwin‐pH‐11B, HORIBA, Kyoto, Japan). The extract was filtered using filter paper 5C (ADVANTEC, Tokyo, Japan) and a syringe filter DISMIC25CS (ADVANTEC). The filtrate was analyzed using an Ion Chromatography system Shim‐pack IC (Shimadzu, Kyoto, Japan) with 0.6 mM Na_2_CO_3_/12 mM NaHCO_3_ as the anion eluent and 2.5 mM oxalic acid as the cation eluent, at a separation column temperature of 40°C. The ion concentrations (Na^+^, ${\mathrm{NH}}_{4}^{+}$, K^+^, Cl^−^, ${\mathrm{NO}}_{3}^{-}$, ${\mathrm{SO}}_{4}^{2-}$, Mg^2+^, and Ca^2+^) were expressed as per 100 g dried substrate bases. A principal component analysis was conducted to visually represent the variance in nutrient ion composition and project it onto PC vectors (Figure S1).

### 2.4 Fungal communities in logs and soil

In September 2020 (for 44 logs and 10 soil quadrats) and September 2021 (for additional 18 logs), three samples were collected from each substrate using a knife and then combined into a single sample (72 combined samples), approximately 30 mL each. The knife was sterilized with 70% ethanol and a burner flame to prevent cross‐contamination among samples. The collected samples were transported back to the laboratory on ice and stored at −30°C until DNA extraction.

DNA extraction was performed using 0.2 g of white and brown rot dead wood and 0.3 g of soil freeze‐dried samples using the ISOIL for Beads Beating kit (Nippon Gene, Tokyo, Japan) following the manufacturer's protocol. Of 72 samples, DNA extraction was unsuccessful for seven samples (three white rot, three brown rot, and one soil sample). The fungal internal transcribed spacer 1 (ITS1) region was sequenced using the MiSeq sequencing platform with 250 × 2 paired‐end reads (Illumina, San Diego, CA, USA). This was done using a two‐step PCR protocol with ITS1F_KYO1/ITS2_KYO2 primers (Toju et al., 2012), where the primary amplification included tails for adding indices and Illumina flow cell adapters in the secondary amplification. We used positive and negative controls in the PCR and positive controls in the MiSeq sequencing. The ITS region is widely recognized as the formal fungal barcode (Kauserud, 2023; Schoch et al., 2012). For more details on sample preparation for MiSeq sequencing, please refer to the Data S1.

A total of 4,004,674 reads were obtained after the MiSeq sequencing run and chimera check. The data were deposited in the Sequence Read Archive of the DNA Data Bank of Japan under the bioproject number PRJDB13698 (DRR532428–DRR532492). After trimming the sequence reads, a minimum quality value of 30 and the 5′‐ and 3′‐primer sequences were subsequently removed from the trimmed reads. Following this, the trimmed reads were denoised using Claident (Tanabe & Toju, 2013) with Assams assembler v0.2.2015.05.10 (Tanabe, 2015). A chimera check was conducted using Claident software concerning the UNITE database (https://unite.ut.ee, accessed October 16, 2023). The quality‐filtered sequences were then classified into molecular operational taxonomic units (OTUs) and taxonomically identified using the Claident fungal ITS database (fungi_its_genus), structured after the International Nucleotide Sequence Database (http://www.insdc.org, accessed December 3, 2019). This classification was performed at a threshold similarity of 97%, a widely recognized standard for the fungal ITS region (Osono, 2014).

We excluded two white rot samples with less than 1000 reads. For each sample, OTUs with below 0.1% of the total number of reads per sample were removed. After filtering, a total of 3,843,172 reads were retained. Since the OTU numbers reached saturation in this dataset (Figure S2), we did not conduct rarefaction for individual samples to adjust for differences in sequence length. Singleton OTUs were also eliminated from subsequent analyses. As a result, each of the 373 filtered OTUs from 63 samples (25 white rot, 29 brown rot, and nine soil samples) was cross‐referenced with the FUNGuild database and assigned to one of the 11 functional groups: arbuscular mycorrhizal (AM), brown rot (Bro), ectomycorrhizal (ECM), ericoid mycorrhizal (Erm), fungal parasite (Fup), plant pathogen (Plp), soft‐rot (Sof), undefined saprotroph (Sap), white rot (Whi), and wood decay with unknown decay type (Wod) and unknown functions (Unk) (https://github.com/UMNFuN/FUNGuild, accessed July 1, 2018; Nguyen et al., 2016) (Table S1).

### 2.5 Colonization rate of mycorrhizal fungi

The colonization rate (%) of mycorrhizal fungi in the root system of individual seedlings was assessed. For AM tree species, one seedling was randomly selected from each quadrat on every substrate (*n* = 9). The roots were washed with a 0.005% aerosol OT solution (Wako, Osaka, Japan) in a Voltex for 1 min, followed by a 10‐min treatment in a hypersonic water bath. Subsequently, they were rinsed with deionized water twice and cleared by heating in 10% KOH at 100°C for over 1 h. After this, the cleared roots were rinsed with deionized water, bleached in 0.5% H_2_O_2_ solution for 20 min, rinsed again with deionized water, and then fixed in 2% HCl for over 10 min. The fixed roots were stained with trypan blue and preserved in lactoglycerol (lactic acid 525 mL, glycerin 37.8 mL, and deionized water 37.2 mL). Colonization was assessed using the method by McGonigle et al. (1990) under 200× magnification to determine the percentage of root length colonized by AM fungal structures, including arbuscules, coils, and vesicles.

For ECM trees species, three seedlings were selected from each quadrat on each substrate (*n* = 27). The colonization rate (%) of ECM fungi was calculated as the percentage of ECM root tips over the total root tips (88.8 tips on average) by directly observing the root systems under a binocular microscope at below 45× magnification (SZ2–ILST, Olympus, Tokyo, Japan).

### 2.6 Statistical analysis

All statistical analyses were performed using R version 4.0.5 (R core team, 2021). To compare the physicochemical properties of the substrates, seed germination rate, seedling survival rate, shoot length, dry weight, and mycorrhizal colonization of seedlings among the three substrates, we employed the Steel–Dwass test with the *nparcomp* command from the *nparcomp* package. Data from *Alnus hirsuta* were excluded from comparison due to very low germination and survival rates.

To visualize the fungal taxonomic composition in the substrates, we utilized the *heat_tree* command from the *metacoder* package (Foster et al., 2017). We based our analysis on the occurrence data (presence/absence) of fungal OTUs, as the numbers of sequence reads do not usually reflect the relative abundance of taxa in a sample (Skelton et al., 2019). To assess the dissimilarities in fungal communities between the samples, we calculated the Raup–Crick index. We created non‐metric multidimensional scaling (NMDS) ordination plots for all surveyed samples using the *metaMDS* command from the *vegan* package (Oksanen et al., 2016). The significance of differences in community composition among the substrates was determined by permutational multivariate analysis of variance (PERMANOVA), with 10,000 permutations, using the *adonis* command (Anderson, 2001). Additionally, the community variance between samples (calculated using the *betadisper* command) was compared among substrates using analysis of variance with the *ANOVA* command. The significance of correlations between environmental variables and fungal community composition was analyzed using the *envfit* command. Among the environmental variables listed in Table S2, we selected canopy openness, pH, water content, and nutrient_PC1 for analysis after removing highly correlated variables to reduce multicollinearity.

Fungal OTU richness at each substrate was compared using iNEXT (Chao et al., 2016). Occurrence frequencies of fungal OTUs belonging to specific functional categories, such as AM and ECM fungi and plant pathogens, were determined as the percentage of logs on which the OTUs of the focal function were detected relative to the total log numbers. We compared the occurrence frequencies of these functional categories among the substrates using Fisher's exact probability test and Ryan's post hoc comparison (http://aoki2.si.gunma‐u.ac.jp/R/src/p_multi_comp.R). These categories were chosen due to their potential effects on seedling growth and mortality (Bayandala & Seiwa, 2016; Wulantuya et al., 2020). Indicator species analysis was applied to the OTUs assigned to functions to determine whether their occurrences indicate specific substrates using the *multipatt* command from the *indicspecies* package (De Cáceres & Legendre, 2009).

Generalized linear models (GLMs) and generalized linear mixed models (GLMMs) were employed to analyze the relationships between environmental variables and seedling performance (germination, survival, and growth). In addition to the five factors that showed significant correlations with fungal communities in the NMDS ordination analysis (substrate, canopy openness, pH, water content, and nutrient_PC1), the OTU richness of AM or ECM fungi was also set as fixed variables. We began by analyzing combined data for AM or ECM trees using GLMM. The OTU richness of AM and ECM fungi was used as factors for models of AM and ECM trees, respectively. For the model explaining germination rate, a matrix of germinated and non‐germinated seed numbers [cbind(germinated, non‐germinated)] in each quadrat was set as the dependent variable. For the model explaining survival rate, a matrix of survived and dead seedling numbers [cbind(survived, dead)] in each quadrat was set as the dependent variable. Binomial distributions (logit link) were assumed, and unmeasured effects of seedling species and sowing year were set as random factors for germination and survival models. For the models explaining seedling growth, shoot length and dried weight of each seedling were set as dependent variables. The shoot length model assumed a Gaussian distribution (identity link), while the dry weight model used a Gamma distribution (log link). The unmeasured effects of substrate identity, seedling species, and sowing year were set as random factors. The best model was selected based on the lowest Akaike information criterion (AIC) using backward elimination in the *dredge* function from the *MuMIn* package (Burnham & Anderson, 2002). For germination and survival models, corresponded AIC (AICc) was used given the small sample size (Burnham & Anderson, 2002).

Subsequently, we analyzed data for individual seedling species using GLM and GLMM using the same sets of variables used in the models for the combined data of mycorrhizal type. For models explaining germination rate and survival rate, we assumed quasi‐Poisson (log link) and quasi‐binomial (logit link) distributions, respectively, due to their significant data variations (Faraway, 2006). As described above, the best models were selected based on the lowest AIC or AICc. Pearson's correlation coefficients among the variables were all below 0.7, and the variance inflation factors of the models were less than 5 (vif function in car package), indicating low levels of multicollinearity.

Pearson's correlation coefficients between the colonization rate of mycorrhizal fungi and seedling growth (shoot length and dry weight) were calculated for each tree species, except for *Alnus* seedlings, from which we could not obtain enough seedlings.

## 3 RESULTS

### 3.1 Physicochemical properties of the substrates

Canopy openness was not significantly different among the three substrates (Figure S3). Brown rot logs had higher water content than white rot logs and soil, whereas the soil pH was higher than that of logs. Among the nutrient ions, concentrations of Na^+^, ${\mathrm{NH}}_{4}^{+}$, and K^+^ were significantly higher in white rot logs than in brown rot logs and soil (Figure S4). In contrast, ${\mathrm{SO}}_{4}^{2-}$ concentration in the soil was higher than in the logs. The Cl^−^, ${\mathrm{NO}}_{3}^{-}$, and Ca^2+^ concentrations were similar across the substrates. ${\mathrm{PO}}_{4}^{3-}$ concentration was too low to detect quantitatively (<0.5 mg/L).

### 3.2 Fungal community in the substrates

**FIGURE 2:**
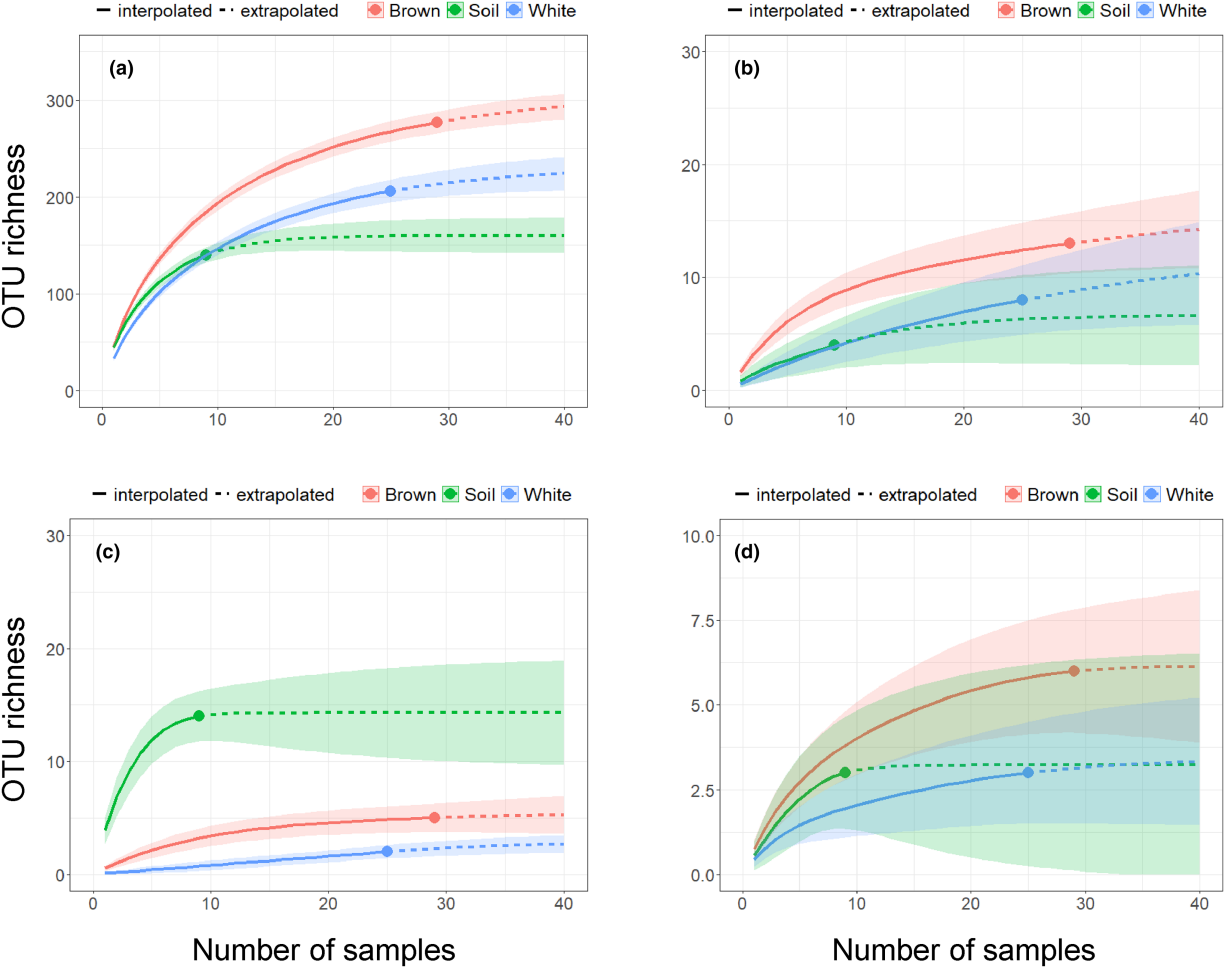
Fungal OTU richness accumulation curves plotted against the number of samples for each substrate. (a) All fungi, (b) arbuscular mycorrhizal fungi, (c) ectomycorrhizal fungi, and (d) plant pathogenic fungi.

In total, 373 OTUs were detected, including 192 Ascomycota, 116 Basidiomycota, 13 Glomeromycota, and nine Mucoromycota, along with 43 unknown taxonomy (Table S1, Figure S5). Leotiomycetes, Sordariomycetes, and Eurotiomycetes were the dominant classes in Ascomycota. Agaricomycetes was the most dominant class among Basidiomycota. Among the 373 OTUs, 109 were assigned to one of the 10 functional groups. Undefined saprotroph (Sap) contained the largest number of OTUs (47 OTUs), followed by ECM (15 OTUs), AM (13 OTUs), Sof (12 OTUs), Whi (7 OTUs), and Plp (7 OTUs). The OTU richness of all fungi was nearly saturated against the number of samples (Figure 2a), with brown rot logs hosting the largest number of fungal OTUs. This was also the case for AM (Figure 2b), while in the ECM group, the highest number of OTUs was found in soil (Figure 2c). Differences in the OTU richness of Plp across the substrates were unclear due to a large overlap in the confidence intervals (Figure 2d).

**FIGURE 3:**
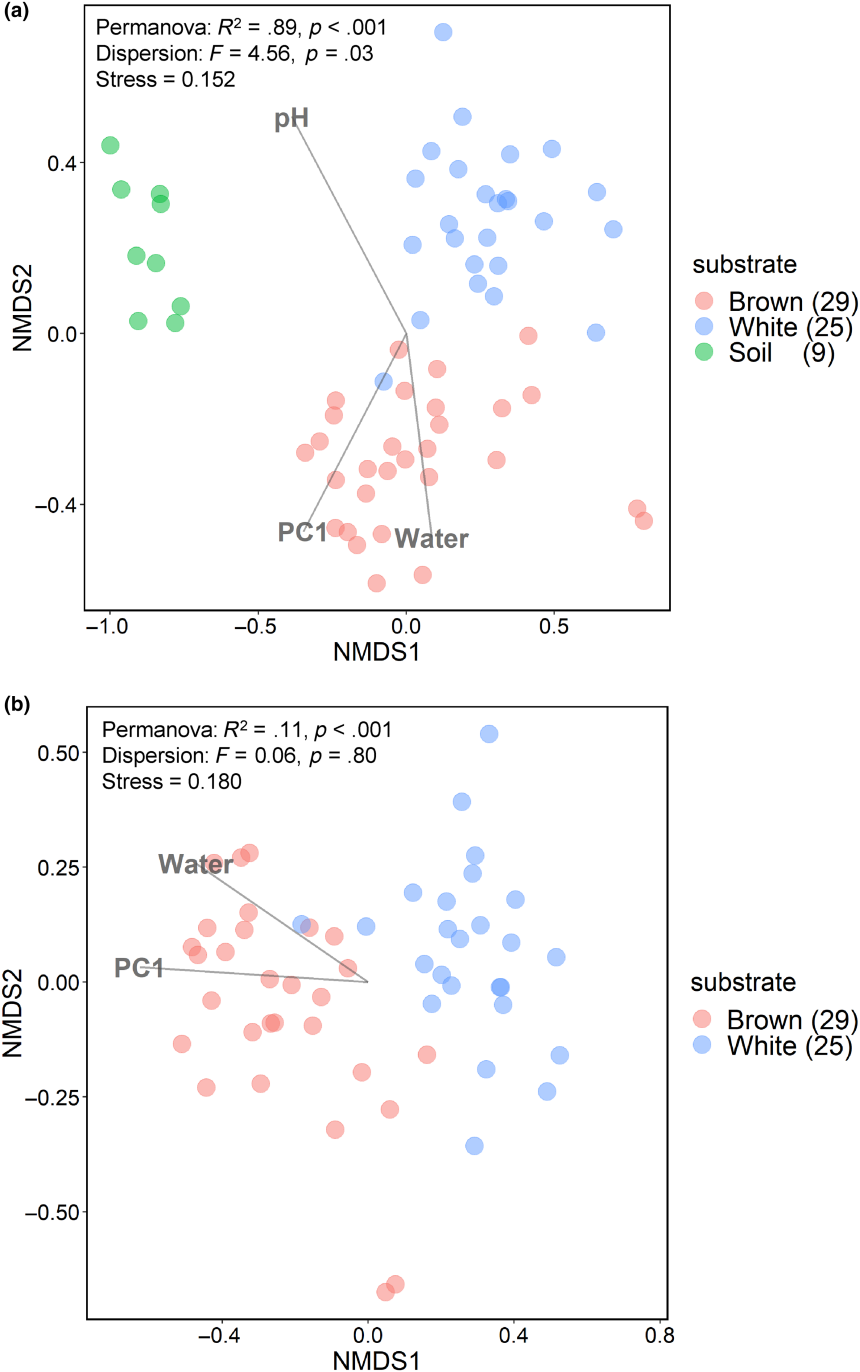
NMDS ordination plot illustrating the dissimilarity of fungal communities in brown and white rot logs and soil samples. (a) Comparison among all three substrates. (b) Comparison between brown and white rot logs. Environmental variables with significant relationships to fungal community structure are displayed as vectors: water, water content; PC1, nutrient_PC1.

NMDS ordination plot and PERMANOVA showed that fungal communities significantly differed (*p* < .001) across the substrates (Figure 3a), although dispersion differed across the substrates (*p* = .03). The sampling year had no effect on the fungal communities (PERMANOVA, *R* ^2^ = .05, *p* = .07). Among the tested environmental variables, pH, water content, and nutrient_PC1 of the substrates had significant associations with fungal communities (Figure 3a). The effects of water content and nutrient_PC1 were consistent. However, the effect of pH was not evident in the dataset focusing on logs (i.e., excluding soil samples) (Figure 3b). The diameter and length of the logs were not significantly associated with fungal communities.

Of the 40 OTUs that indicated the three substrates (Table 2), the number of indicative OTUs for brown and white rot logs and soil were 34, 22, and 76, respectively. All substrates included indicative Sap OTUs. In contrast, all 3 AM OTUs were found in brown rot samples, and all 12 ECM OTUs were found in soil samples. An indicative Bro OTU (*Leucogyrophana* sp.) and a Whi OTU (*Sistotremastrum* sp.) were found in brown and white rot samples, respectively.

**TABLE 2:** Fungal OTUs indicating each of the three substrates.

OTU_ID	Substrate	Function	Taxa	Stat	*p*
OTU_1171	Brown	AM	Glomeraceae sp.	0.358	.0284
OTU_2226	Brown	AM	Glomeraceae sp.	0.358	.0263
OTU_1653	Brown	AM	*Glomus* sp.	0.349	.0418
OTU_3243	Brown	Bro	*Leucogyrophana* sp.	0.349	.0436
OTU_925	Brown	Sap	*Mortierella* sp.	0.696	.0001
OTU_1080	Brown	Sap	*Mortierella* sp.	0.569	.0006
OTU_799	Brown	Sap	*Hyphoderma* sp.	0.48	.0021
OTU_2641	Brown	Sap	*Mollisia* sp.	0.412	.0142
OTU_1259	Brown	Sap	*Mollisia* sp.	0.358	.0253
OTU_2899	Brown	Sof	*Scytalidium* sp.	0.385	.0138
OTU_1595	White	Sap	*Sugiyamaella* sp.	0.737	.0001
OTU_1827	White	Sap	*Hyaloscypha* sp.	0.489	.0013
OTU_3258	White	Sap	*Stypella vermiformis*	0.361	.0354
OTU_1473	White	Whi	*Sistotremastrum* sp.	0.775	.0001
OTU_1135	White	Wod	*Botryobasidium* sp.	0.546	.0017
OTU_3033	Soil	ECM	*Russula* sp.	0.674	.0001
OTU_1999	Soil	ECM	*Tomentella* sp.	0.59	.0004
OTU_41	Soil	ECM	*Elaphomyces* sp.	0.5	.0013
OTU_51	Soil	ECM	*Tomentella* sp.	0.5	.0021
OTU_1717	Soil	ECM	*Russula* sp.	0.5	.002
OTU_316	Soil	ECM	*Russula* sp.	0.413	.0066
OTU_246	Soil	ECM	*Thelephora* sp.	0.4	.0199
OTU_303	Soil	ECM	*Tomentella* sp.	0.4	.0174
OTU_1056	Soil	ECM	*Sarcodon* sp.	0.4	.0178
OTU_2514	Soil	ECM	*Rhizoscyphus* sp.	0.4	.0196
OTU_2861	Soil	ECM	*Thelephora* sp.	0.4	.0172
OTU_3027	Soil	ECM	*Russula* sp.	0.345	.0288
OTU_2845	Soil	Erm	*Oidiodendron* sp.	0.5	.0013
OTU_1785	Soil	Plp	*Colletotrichum* sp.	0.4	.0169
OTU_2323	Soil	Sap	*Umbelopsis* sp.	0.918	.0001
OTU_2063	Soil	Sap	*Mortierella* sp.	0.886	.0001
OTU_3112	Soil	Sap	*Geminibasidium* sp.	0.886	.0001
OTU_529	Soil	Sap	*Sagenomella* sp.	0.837	.0001
OTU_2733	Soil	Sap	*Mortierella* sp.	0.713	.0002
OTU_2019	Soil	Sap	*Saitozyma podzolica*	0.686	.0001
OTU_3025	Soil	Sap	*Cladophialophora* sp.	0.674	.0002
OTU_1451	Soil	Sap	*Penicillium* sp.	0.4	.0171
OTU_189	Soil	Sap	*Cladophialophora* sp.	0.345	.0284
OTU_139	Soil	Sof	*Trichoderma* sp.	0.583	.0003
OTU_2831	Soil	Sof	*Trichoderma* sp.	0.4	.0176

### 3.3 Seedling demography

**FIGURE 4:**
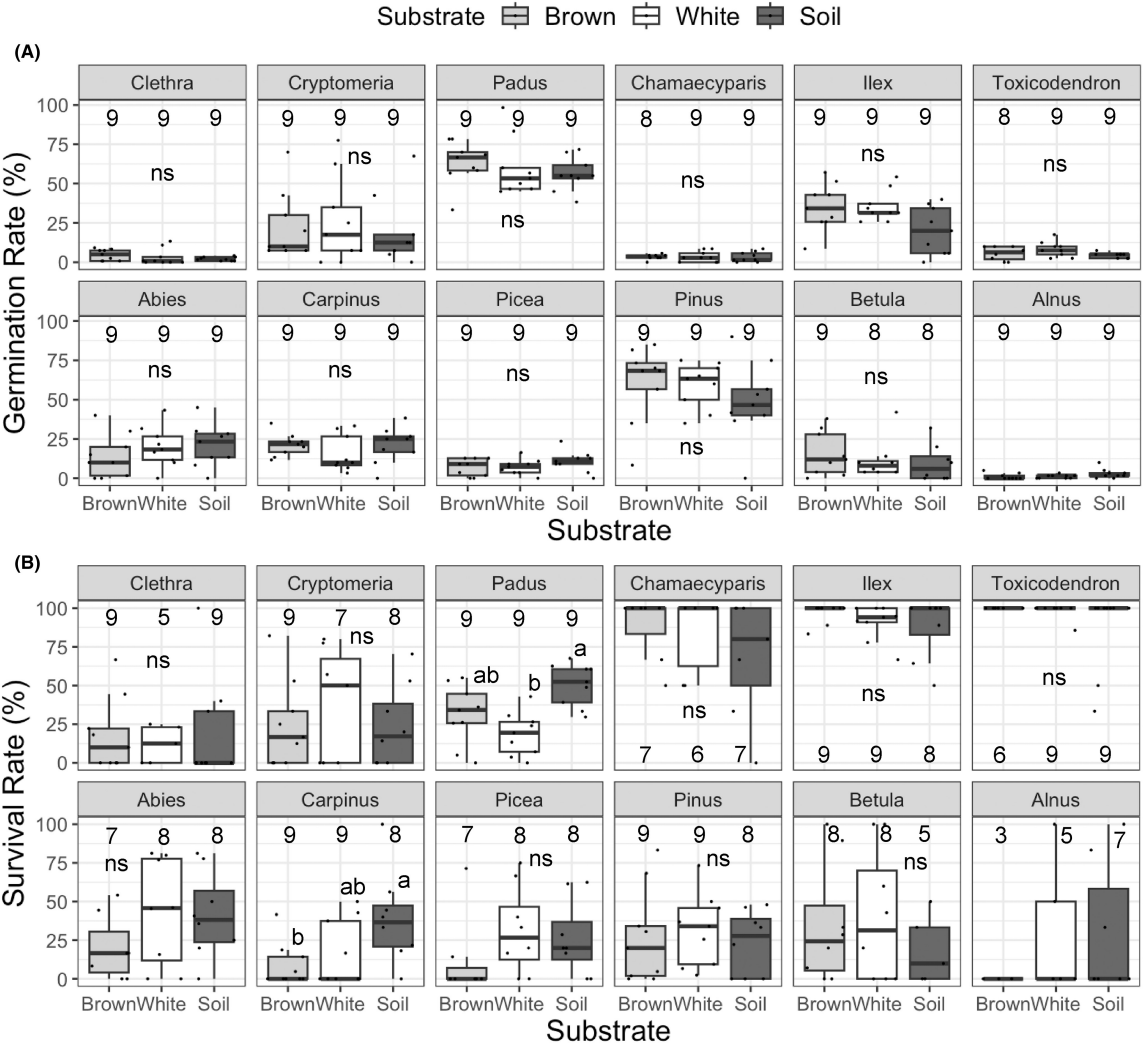
Germination (A) and survival (B) rate of 12 tree species on brown and white rot logs and soil. Different lowercase letters on the boxes indicate significant differences across the substrates (Steel–Dwass test, *p* < .05), while ns indicate no significant difference (Steel–Dwass test, *p* > .05). The numbers indicate the number of replicates, which was originally nine but was reduced in some cases due to accidental disturbance by animals. Upper row: arbuscular mycorrhizal trees. Bottom row: ectomycorrhizal trees.

Of the 19,170 seeds sown, 3562 (18.6%) seeds germinated. Of the 12 tree species tested, *Padus grayana* and *Pinus densiflora* showed germination rates of >50% on all substrates (Figure 4A). The germination rates of *Ilex crenata*, *Cryptomeria japonica*, *Abies veitchii*, *Carpinus laxiflora*, and *Betula ermanii* were 20%–30%. However, *Chamaecyparis obtusa*, *Toxicodendron trichocarpa*, *Picea jezoensis*, *Clethra barbinervis*, and *Alnus hirsuta* showed germination rates of <10%. The data on *Alnus hirsuta* were not included in the following analysis due to its low germination rate. None of the 12 tree species showed a significant difference in germination rate across the three substrates. The name of the seedling species will be represented by their genus names hereafter.

Among the seeds sown in November 2019, *Abies*, *Carpinus*, and *Padus* had germinated by the first recording on April 16, 2020. The populations decreased gradually during the first growing season and remained constant thereafter, even after winter (Figure S6a). The germination of *Cryptomeria*, *Clethra*, *Pinus*, and *Picea* peaked in June, and their population decreased during summer but remained constant thereafter, even after winter. Among the seeds sown in November 2020, *Chamaecyparis*, *Ilex*, and *Toxicodendron* showed high survival rates (Figure S6b, Figure 4B). *Betula* germinated from April to May and decreased its population to approximately 50% of the original germinants by autumn (Figure S6b).

**FIGURE 5:**
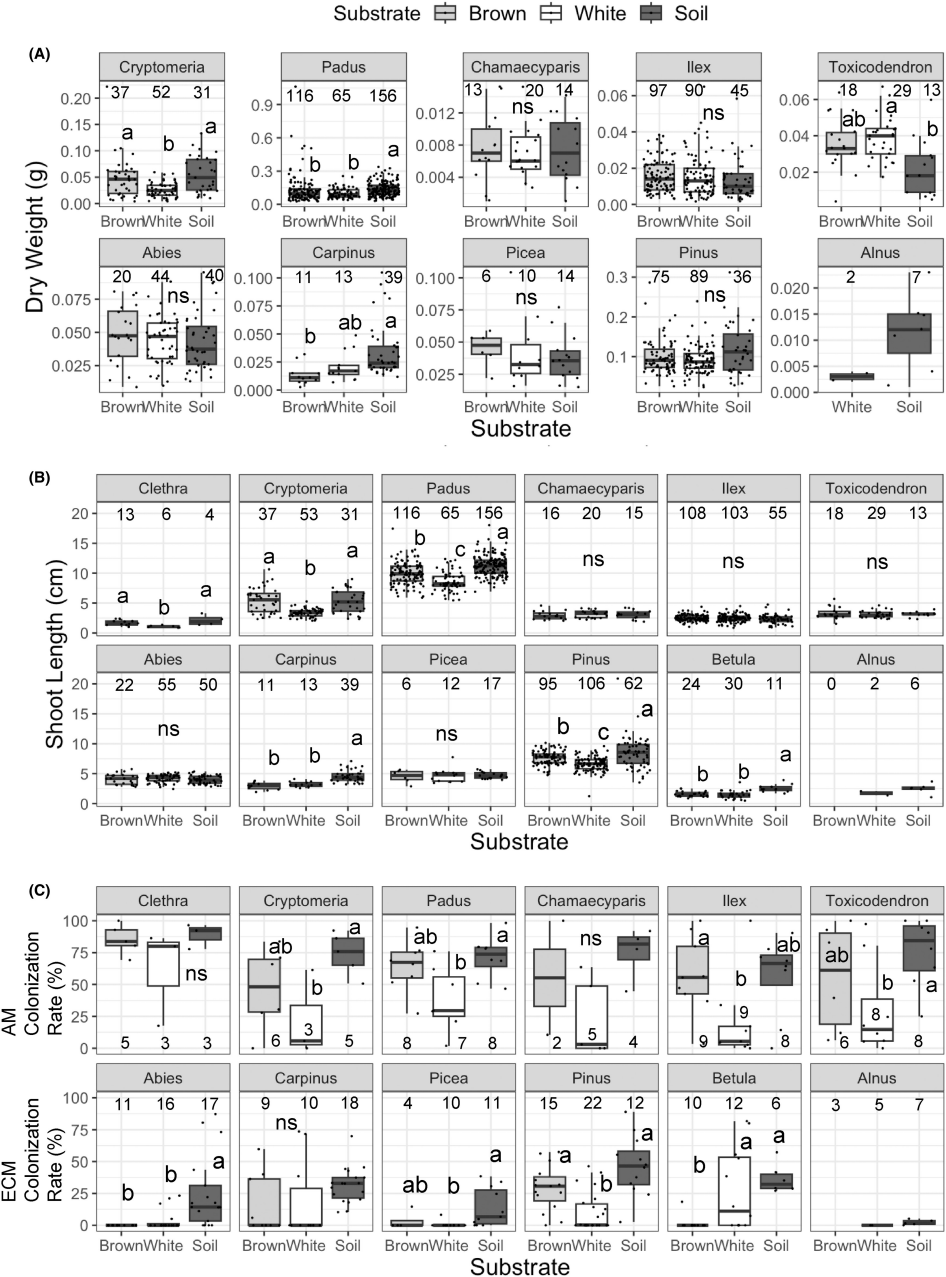
The dry weight of the entire seedling (A), shoot length (B), and colonization rate of mycorrhizal fungi (C) of the tested tree species on brown and white rot logs and soil. Different lowercase letters on the boxes indicate a significant difference across the substrates (Steel–Dwass test, *p* < .05). Ns indicates no significant differences across the three substrates (Steel–Dwass test, *p* > .05). The numbers indicate the number of replicate seedlings used for the measurements. Upper row: arbuscular mycorrhizal trees. Bottom row: ectomycorrhizal trees. Dry weight was not measured for *Clethra* and *Betula* seedlings because they were too lightweight.

At the time of seedling harvest in October 2021, 32.7% and 75.8% of the seedlings sown in November 2019 and November 2020 survived, respectively, resulting in a total of 1422 seedlings harvested. *Padus* and *Carpinus* seedlings showed significantly lower survival rates on white and brown rot logs, respectively, compared to soil (Figure 4B). The dry weight of *Cryptomeria* seedlings was larger on brown rot logs and soil compared to white rot logs (Figure 5A). While the dry weights of *Padus* and *Carpinus* seedlings were larger on soil compared to logs, the opposite was seen for *Toxicodendron* seedlings. The shoot lengths of *Clethra*, *Cryptomeria*, *Padus*, and *Pinus* seedlings were longer on brown rot logs and soil compared to white rot logs (Figure 5B), while those of *Carpinus* and *Betula* seedlings were greater on soil compared to logs. The colonization rate of AM fungi was generally high among all the six AM tree species tested and tended to be lower on white rot logs compared to brown rot logs and soil, although the difference between brown rot and white rot logs was not significant, except for *Ilex* (Figure 5C). The colonization rate of ECM fungi was higher on soil compared to logs in *Abies* and *Picea* seedlings. *Pinus* seedlings showed a significantly higher colonization rate of ECM fungi on brown rot logs and soil than on white rot logs. In contrast, the colonization rate of ECM fungi was significantly higher in *Betula* seedlings growing on white rot logs and soil than in brown rot logs. The colonization rate of ECM fungi in *Carpinus* seedlings was not significantly different across the substrates.

### 3.4 Factors regulating seedling performance

The GLMM results indicated that substrate was selected in the model, explaining the germination rate, survival rate, and shoot length of AM tree species and the survival rate of ECM tree species (Table 3). The germination rate of AM trees was significantly larger on white rot logs and soil than on brown rot logs (Table 4). The survival rate of AM trees was significantly higher on soil than on brown rot and white rot logs. The shoot length of AM trees was significantly larger on soil than on white rot logs and was marginally (*p* < .1) larger on brown rot logs than on white rot logs. The survival rate of ECM trees was significantly higher on white rot logs and soil than on brown rot logs. The water content of the substrates was commonly selected as a positive factor for the germination and survival of both AM and ECM trees (Table 3). Canopy openness was selected as a positive factor for the survival rate of ECM trees but as a negative factor for the germination of both AM and ECM trees.

**TABLE 3:** GLMM results illustrate the relationships among germination rate, survival rate, dry weight, and shoot length of seedlings from arbuscular mycorrhizal (AM) and ectomycorrhizal (ECM) tree species and environmental factors.

Mycorrhizal type	Seedling performance	*n*	Variables					
			Substrate	PC1	pH	Water	Openness	OTU richness^a^
AM trees	Germination	146	SIG*	−0.0039***	−0.3781**	0.0699***	−0.0386***	0.0346
	Survival	130	SIG*	−0.0029		0.0818***		
	Dry weight	689					−0.0014	
	Shoot length	738	SIG*					
ECM trees	Germination	146			0.3492***	0.0371***	−0.0328***	
	Survival	123	SIG*			0.0747***	0.0573***	
	Dry weight	378					0.0262	
	Shoot length	529			0.6706			

Abbreviation: PC1, nutrient_PC1.

^a^ OTU richness of mycorrhizal fungi: AM fungal OTU richness for AM trees, and ECM fungal OTU richness for ECM trees.

*p < .05; **p < .01; ***p < .001. SIG, significant effect.

**TABLE 4:** Multiple comparisons among the estimates for arbuscular mycorrhizal (AM) and ectomycorrhizal (ECM) tree seedling performance concerning the substrates where the relationships with substrates were selected as a factor in the GLMM in this table.

Mycorrhizal type	Seedling performance	Substrate combination	Estimate^a^	SE	Z	*p*
AM	Germination	White–Brown	0.3044	0.1129	2.697	.019
		Soil–Brown	0.5136	0.1276	4.024	<.001
		Soil–White	0.2091	0.1184	1.766	.18
	Survival rate	White–Brown	−0.1022	0.2009	−0.509	.867
		Soil–Brown	1.3022	0.1907	6.828	<.001
		Soil–White	1.4044	0.1897	7.405	<.001
	Shoot length	White‐Brown	−0.6088	0.2875	−2.118	.085
		Soil–Brown	0.6001	0.3521	1.705	.201
		Soil–White	1.2089	0.3641	3.32	.003
ECM	Survival rate	White–Brown	0.9066	0.1436	6.315	<.001
		Soil–Brown	1.1842	0.1774	6.676	<.001
		Soil–White	0.2776	0.1454	1.909	.134

^a^ When the substrate combination White–Brown was estimated to be positive, white rot logs positively affected the seedlings compared to brown rot logs.

GLMs (for germination and survival) and GLMMs (for dry weight and shoot length) for each tree species indicated that the relationships between seedling performance and environmental factors varied among tree species. The substrate had a significant association with the survival rates of *Cryptomeria*, *Padus*, and *Carpinus*, the dry weight of *Cryptomeria*, and the shoot length of *Padus* (Table S3). The dry weight of *Cryptomeria* seedlings was larger on brown rot logs than on white rot logs (Table S4). The survival rate of *Cryptomeria* seedlings was larger on soil than on brown rot logs. The survival rate and shoot length of *Padus* seedlings were larger on soil than on brown and white rot logs. Among the environmental factors, the water content of the logs had the most widespread association with seedling performance, except for *Chamaecyparis*, *Ilex*, and *Picea* (Table S3). While the associations were mostly positive, shoot length was negatively associated with the water content of the logs in the case of *Carpinus* seedlings. The association with canopy openness varied among seedling species and performance. The dry weights of *Chamaecyparis*, *Ilex*, *Abies*, and *Pinus* were positively associated with canopy openness. Similarly, the shoot length of *Chamaecyparis*, germination of *Picea*, and the survival of *Pinus* showed positive associations with canopy openness. However, the germination of *Clethra* and *Toxicodendron* and the survival of *Abies* were negatively associated with canopy openness. The pH of the logs was positively associated with the dry weight of *Abies* and *Pinus*, the germination of *Picea*, and the shoot length of *Pinus* but negatively with the germination of *Ilex*. The OTU richness of mycorrhizal fungi had negative associations with the dry weight of *Cryptomeria* and the germination of *Picea*. Nutrient_PC1 did not have any significant associations with seedling performance.

**FIGURE 6:**
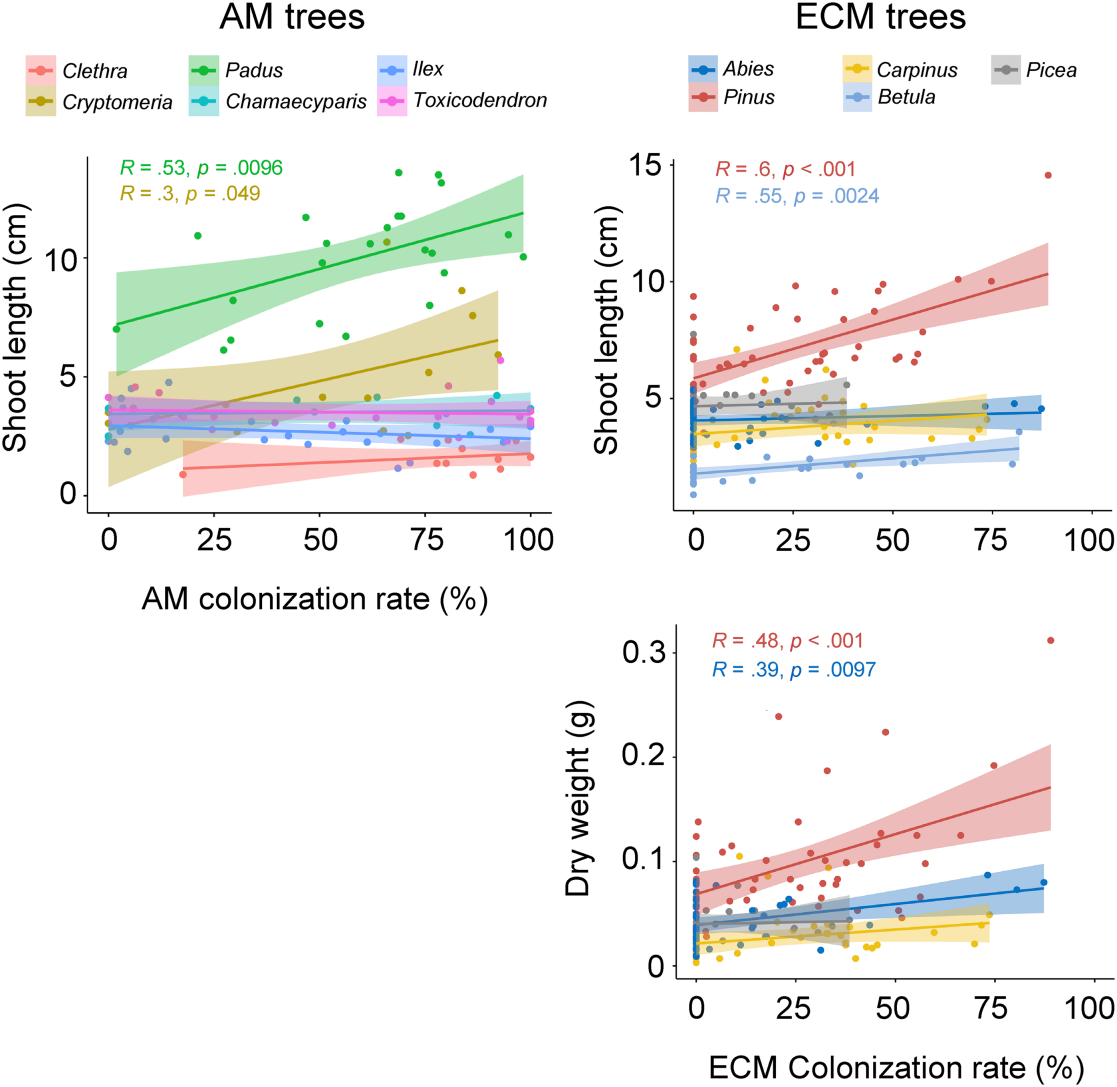
Relationships between seedling performance (shoot length and dry weight) and the colonization rate of mycorrhizal fungi. Left: seedlings of arbuscular mycorrhizal (AM) trees. Right: seedlings of ectomycorrhizal (ECM) trees. If the relationship was statistically significant (*p* < .05), Pearson's correlation coefficient (*R*) and *p*‐value were displayed in the figure. As the relationships between the dry weight of AM trees and the colonization of AM fungi were not significant, they were not shown.

In the seedlings of *Cryptomeria*, *Padus*, *Pinus*, and *Betula*, significantly positive correlations were found between their shoot length and mycorrhizal colonization rate (Figure 6). Similarly, the dry weight of *Abies* and *Pinus* seedlings and their ECM colonization rate exhibited significant positive correlation. The dry weights of none of the AM tree seedlings showed significant relationships with their AM colonization rate.

## 4 DISCUSSION

The present study revealed that the difference in wood decay type affects the fungal community in logs (Figure 3). Particularly, the OTU richness of AM fungi was significantly larger in brown rot than in white rot logs (Figure 2), supporting our hypothesis. However, the reason for this might not be due to the weak competitive exclusion in brown rot logs as we initially hypothesized because the OTU richness of all fungi and that of ECM fungi were also higher in brown rot logs than white rot logs (Figure 3). Instead, the higher water content in brown rot logs compared to white rot logs might have positive effects on fungal OTU richness because high water content is important for hosting diverse fungal communities (Pouska et al., 2017). The water absorption capacity of wood decayed by brown rot fungi significantly increases (Karppanen et al., 2008), which might be beneficial for the plant roots growing into the logs from the ground and also for the development of surface vegetation and seedling establishment, which will be discussed later. The pre‐existing plant roots in the logs strongly affect the mycorrhizal communities in the logs. Herein, *P. densiflora* logs belonging to decay class IV were used, which usually have some vegetation on the top. In the case of logs with brown rot, the vegetation was dominated by AM trees, such as *Cryptomeria japonica* (Fukasawa et al., 2017) and *Clethra barbinervis* (Fukasawa, 2012). The dominance of AM trees induces the dominance of AM fungi belowground (Sawada et al., 2023), which, in turn, benefits the successful establishment of AM tree seedlings (Seiwa et al., 2020).

The difference in wood decay type also affected the seedling performance: germination of AM tree seedlings and the survival of ECM tree seedlings (Table 4). However, in the cases of AM trees, the relationship observed between germination rate and wood decay type was opposite to our hypothesis, which predicts better performance of AM tree seedlings on brown rot logs. Also, none of the specific AM tree species showed a significant difference in germination rate between brown and white rot logs (Figure 4; Table S3). Instead, the shoot length of the seedlings of *Clethra*, *Cryptomeria*, and *Padus* was significantly larger on brown rot logs than on white rot logs. Similarly, the dry weight of *Cryptomeria* seedlings was significantly larger on brown rot logs than on white rot logs (Figure 5). The shoot length of *Cryptomeria* and *Padus* seedlings was positively associated with the colonization rate of AM fungi on their roots (Figure 6), which tended to be higher in brown rot logs than white rot logs (Figure 5). Therefore, the enhanced growth of these seedlings on brown rot logs might be partly due to the high colonization rate of AM fungi on the roots of these seedlings. Colonization with AM fungi is essential for nutrient acquisition, particularly in substrates with poor nutrient contents, such as decaying logs (Fukasawa, 2012), and for protection against pathogens (Filion et al., 1999). Similarly, a previous study using laboratory pot experiments with wood substrates from other forest sites showed that the AM colonization rate on *Cryptomeria* seedlings in brown rot was higher than that on white rot wood (Fukasawa & Kitabatake, 2022). The high water content of brown rot logs (Figure 2) might further enhance seedling performance as water content has been shown to positively affect seedling performance regardless of their mycorrhizal status (Table S3).

In contrast to the better performance of *Clethra*, *Cryptomeria*, and *Padus* on brown rot logs than on white rot logs, the performance of other AM tree seedlings (*Chamaecyparis*, *Ilex*, and *Toxicodendron*) was not significantly different between brown and white rot logs (Figures 4 and 5) despite the significantly higher AM colonization rate of *Ilex* on brown rot logs than white rot logs (Figure 5). Since the AM colonization rate of *Chamaecyparis*, *Ilex*, and *Toxicodendron* did not have significant relationships with their growth (shoot length, Figure 6), their performance might be driven by other factors, such as canopy openness (Table S3), that were not significantly different across the substrates (Figure S3).

In the cases of ECM tree seedlings, we detected a positive effect of white rot on seedling survival of ECM trees compared with brown rot (Table 4), although a significant difference was not observed in any particular ECM trees (Figure 4). Furthermore, the colonization rate of ECM fungi was not higher on white rot logs than on brown rot logs, except in case of *Betula* (Figure 5). Rather, it was higher on brown rot logs than white rot logs in *Pinus* seedlings, which might reflect the OTU richness of ECM fungi in the three substrates (Figure 2). Similarly, the growth of *Pinus* seedlings was significantly correlated with colonization rate of ECM fungi (Figure 6), indicating that mycorrhizal colonization is crucial for the growth of *P. densiflora* seedlings as reported previously (Sim & Eom, 2006). However, the reason why the colonization rate of ECM fungi on *Betula* seedlings was higher on white rot logs than on brown rot logs was not clear and might be possibly due to specific ECM fungi colonized on *Betula* seedlings on white rot logs. Tedersoo et al. (2008) reported that the colonization frequency of an ECM fungus *Amphinema byssoides* was significantly higher in the roots of *Betula pendula* seedlings on white rot logs than brown rot logs. Similarly, Nara (2006) reported that the colonization frequency of the ECM fungus *Tomentella* sp. was significantly higher in *Betula ermanii* seedlings than in other tree seedlings, such as *Larix* and *Salix*, in a mountain volcanic desert. However, in the present study, *Russula* sp. and *Lactarius* sp. were the only ECM fungal taxa detected on white rot logs, and their occurrence frequencies were not particularly high on white rot logs (Table S1).

All 12 tree species used in the present study frequently regenerate as seedlings on decaying logs (Fukasawa, 2021). However, we observed that none of the tree species showed better performance on the logs (regardless of brown or white rot) compared to the soil, which might be due to the high colonization rate of mycorrhizal fungi in the soil (Figure 5). In addition, certain factors that prevent germination and growth on the ground were reduced in this study. For example, the accumulated thick litter layer on the ground, which prevents the germination and growth of small seedlings, was removed, and bare soil was prepared for the seeds. In addition, the light condition (canopy openness) was not significantly different among the microsites. Although the water content of the soil was significantly lower than that of brown rot logs, the high colonization rate of mycorrhizal fungi might enhance the water‐absorbing ability of seedling roots through their mycelial network (Lehto & Zwiazek, 2011). Furthermore, the OTU richness of plant pathogenic fungi was not significantly different among the three microsites (Figure 2), and the negative effects of these fungi on the seedling performance on soil (Cheng & Igarashi, 1987; O'Hanlon‐Manners & Kotanen, 2004) were not observed in the present study. This might be partly attributable to the dry soil of the study site. Consistent with previous studies, the concentrations of nutrients, such as potassium and chloride ions, were higher in white rot logs (Fukasawa et al., 2017; Ostrofsky et al., 1997; Takahashi et al., 2000) than in brown rot logs and soil, although these differences did not significantly affect the seedling performance. These results suggest that the difference in nutrient condition among brown and white rot logs and soil might not be the primary factor determining seedling performance on those substrates in this study site.

The present study comprehensively evaluated the abiotic and biotic factors associated with decaying logs of different decay types and soil in relation to tree seedling regeneration. The results indicated that the three substrates (brown and white rot logs and soil) harbor significantly distinct fungal communities, which partly supported our first hypothesis. The seedling performance of some of the tested 12 tree species showed significant associations with the decay types of logs, possibly due to the difference in symbiotic mycorrhizal fungal communities and water content. However, these associations were seedling species dependent and not consistent across the seedling species in the same mycorrhizal type, which did not support our second hypothesis. Further studies should be conducted using more tree species to evaluate seedling performance on logs with different decay types in field and laboratory experiments.

## AUTHOR CONTRIBUTIONS

**Yu Fukasawa:** Conceptualization (lead); data curation (lead); formal analysis (equal); funding acquisition (lead); investigation (lead); methodology (lead); project administration (lead); resources (lead); supervision (lead); validation (equal); visualization (equal); writing – original draft (lead); writing – review and editing (lead). **Hiroyuki Kitabatake:** Formal analysis (equal); investigation (equal); methodology (equal); writing – original draft (equal); writing – review and editing (equal).

## CONFLICT OF INTEREST STATEMENT

The authors declare that the research was conducted in the absence of any commercial or financial relationships that could be construed as a potential conflict of interest. Experimental research and field studies on fungi including the collection of fungal material are complied with relevant institutional, national, and international guidelines and legislation.

## Supporting information


Figures S1–S6:

Tables S1–S4:

Data S1:

Data S2:

Data S3:

## Data Availability

The data presented in this study are available as supplementary files.
