# Supplementary material for: Factors associated with seedling establishment on logs of different fungal decay types—A seed‐sowing experiment
Source: Ecol Evol. 2024 Jun 4;14(6):e11508. doi: 10.1002/ece3.11508 (PMC11148398; doi:10.1002/ece3.11508)
Supplement: Supplementary file 1 — Figures S1–S6: [file ECE3-14-e11508-s001.pdf]

Supplementary Figures

Factors associated with seedling establishment on logs of different fungal decay types – a seed sowing experiment

Yu Fukasawa*, Hiroyuki Kitabatake

Laboratory of Forest Ecology, Graduate School of Agricultural Science, Tohoku University, 232-3 Yomogida, Naruko, Osaki, Miyagi 989-6711, Japan

*Corresponding author
Email: yu.fukasawa.d3@tohoku.ac.jp, Tel: +81 229 84 7397, Fax: +81 229 84 6490

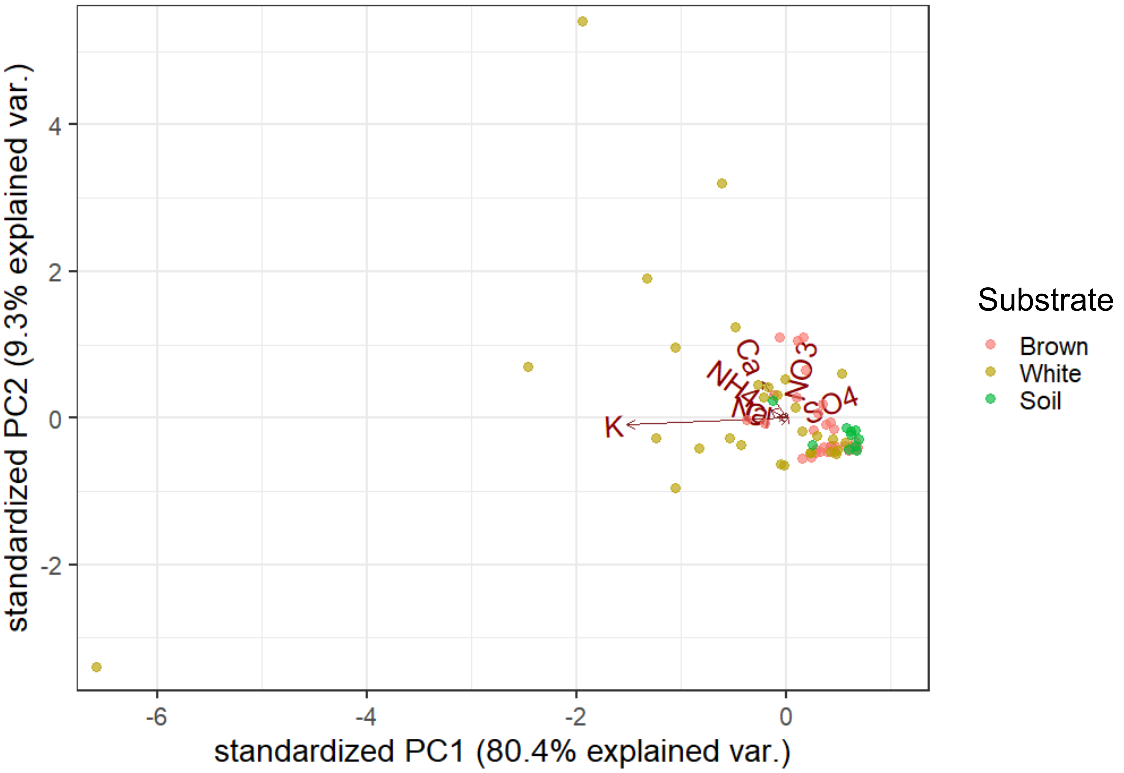

Fig. S1 Principal component analysis of nutrient ions of the substrates. Axis 1 explained 80.4% of the variation.

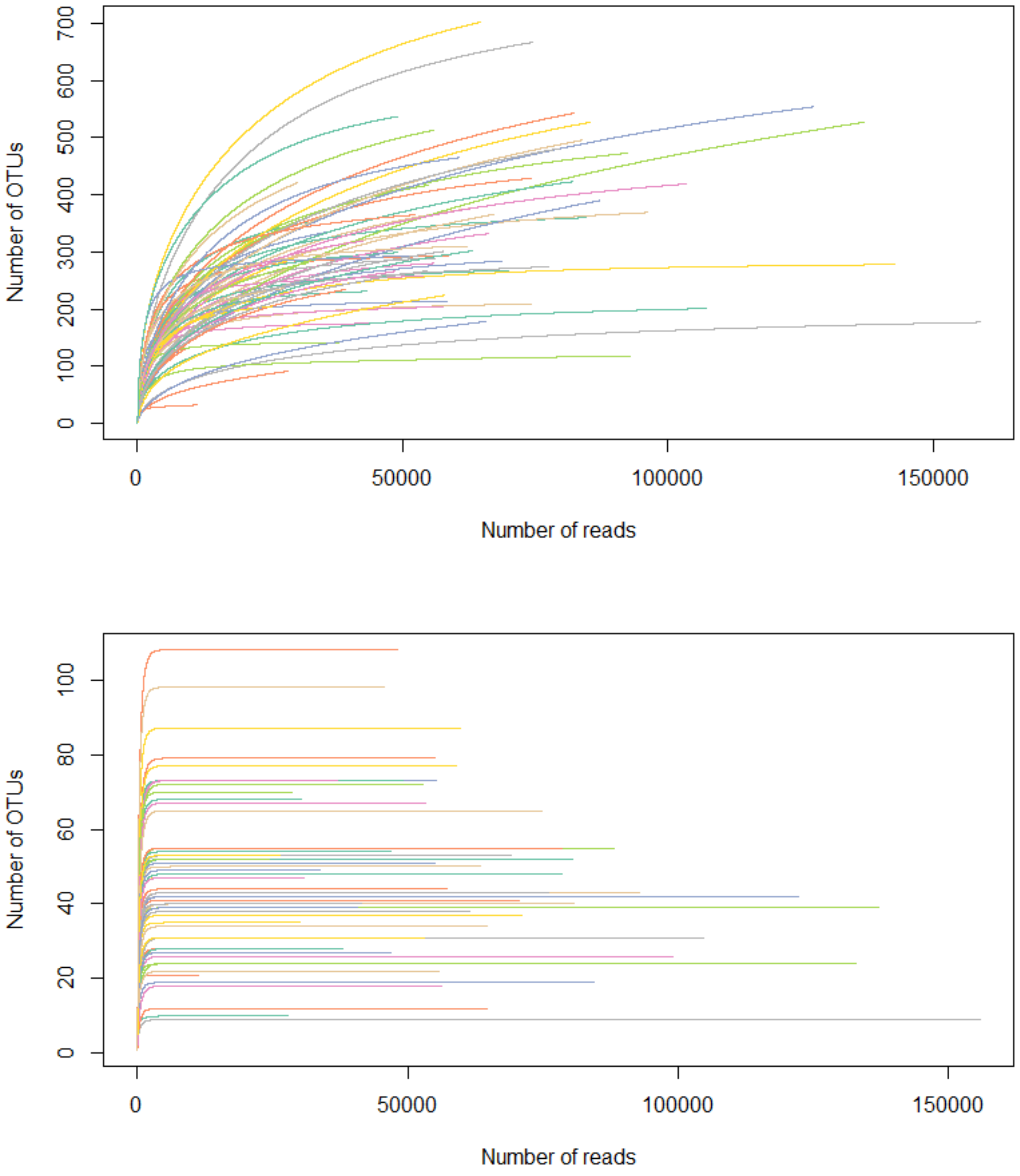

Fig. S2 Sample rarefaction curves for OTU richness plotted against the number of reads detected in the sample before (A) and after (B) the removal of OTUs with less than 0.1% of the total number of reads per sample.

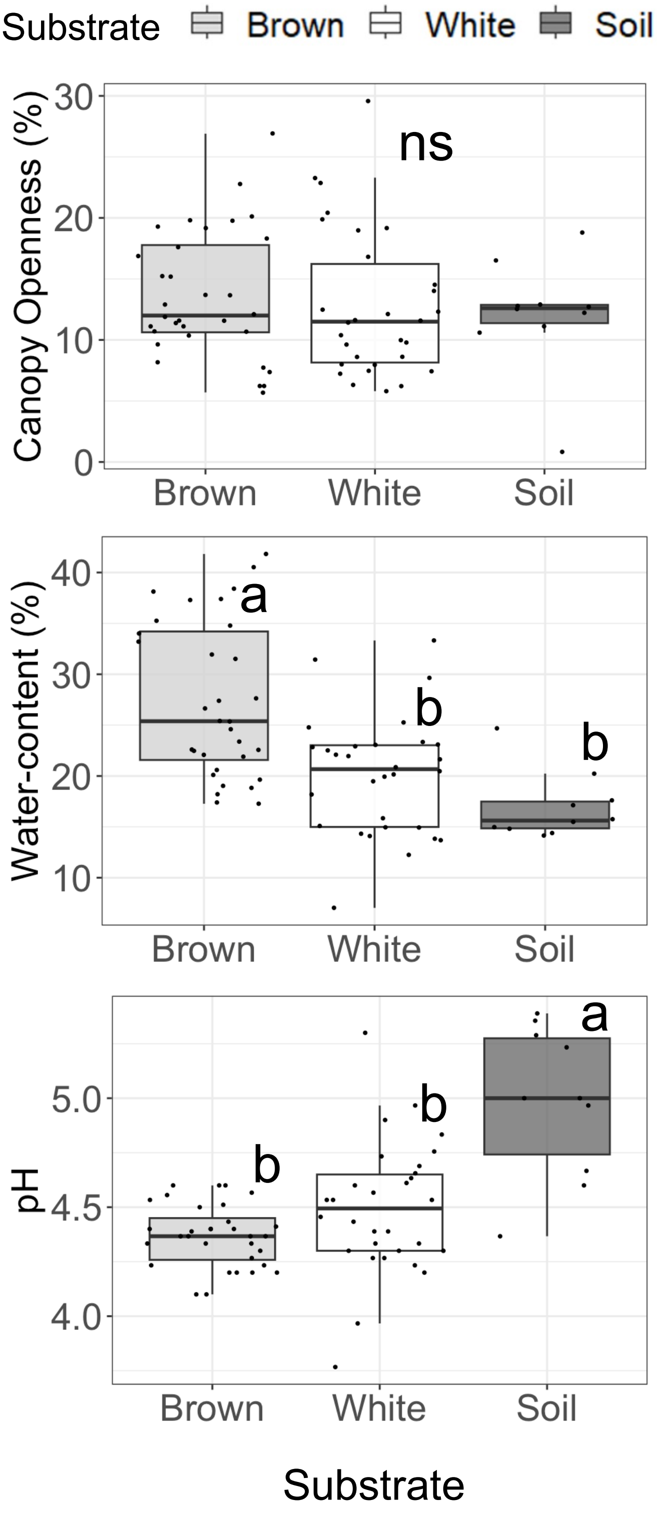

Fig. S3 Physicochemical properties of the substrates: brown rot logs (n = 32); white rot logs (n = 30); soil (n = 10). Different letters indicate a significant difference between the substrates (Steel-Dwass test, $P < 0.05$). Ns indicate no significant differences across the three substrates (Steel-Dwass test, $P > 0.05$).

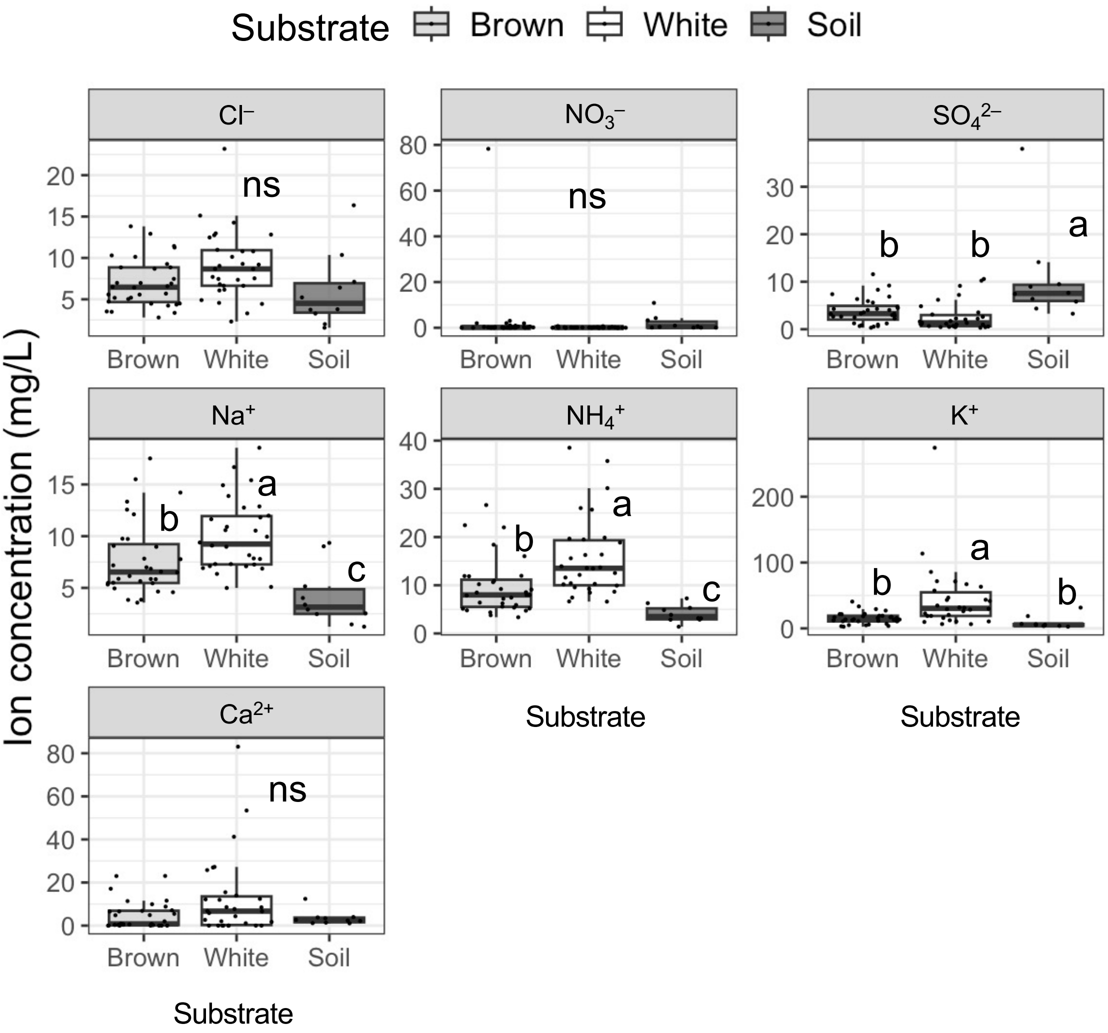

Fig. S4 Nutrient ion concentrations of the substrates: brown rot logs (n = 32); white rot logs (n = 30); soil (n = 10). Different letters indicate a significant difference between the substrates (Steel-Dwass test, $P < 0.05$). Ns indicate no significant differences across the three substrates (Steel-Dwass test, $P > 0.05$).

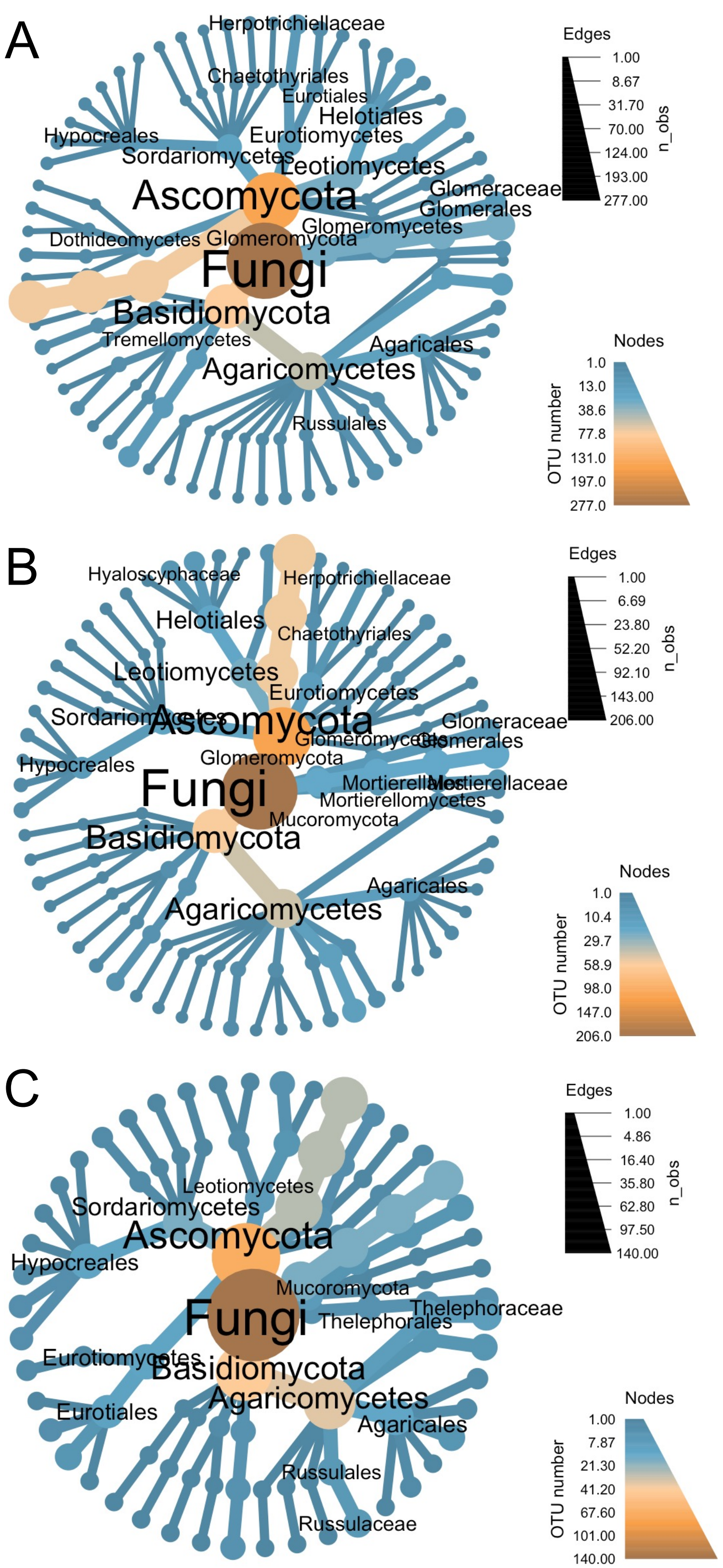

Fig. S5 Taxonomic composition of fungal communities in brown rot (A) and white rot (B) *Pinus densiflora* logs and in soil (C), displayed from Kingdom to Family levels. The colour and size of the tree nodes represent the number of OTUs for that taxon after removing singleton OTUs. Taxon names are displayed for nodes with more than 5 observations.

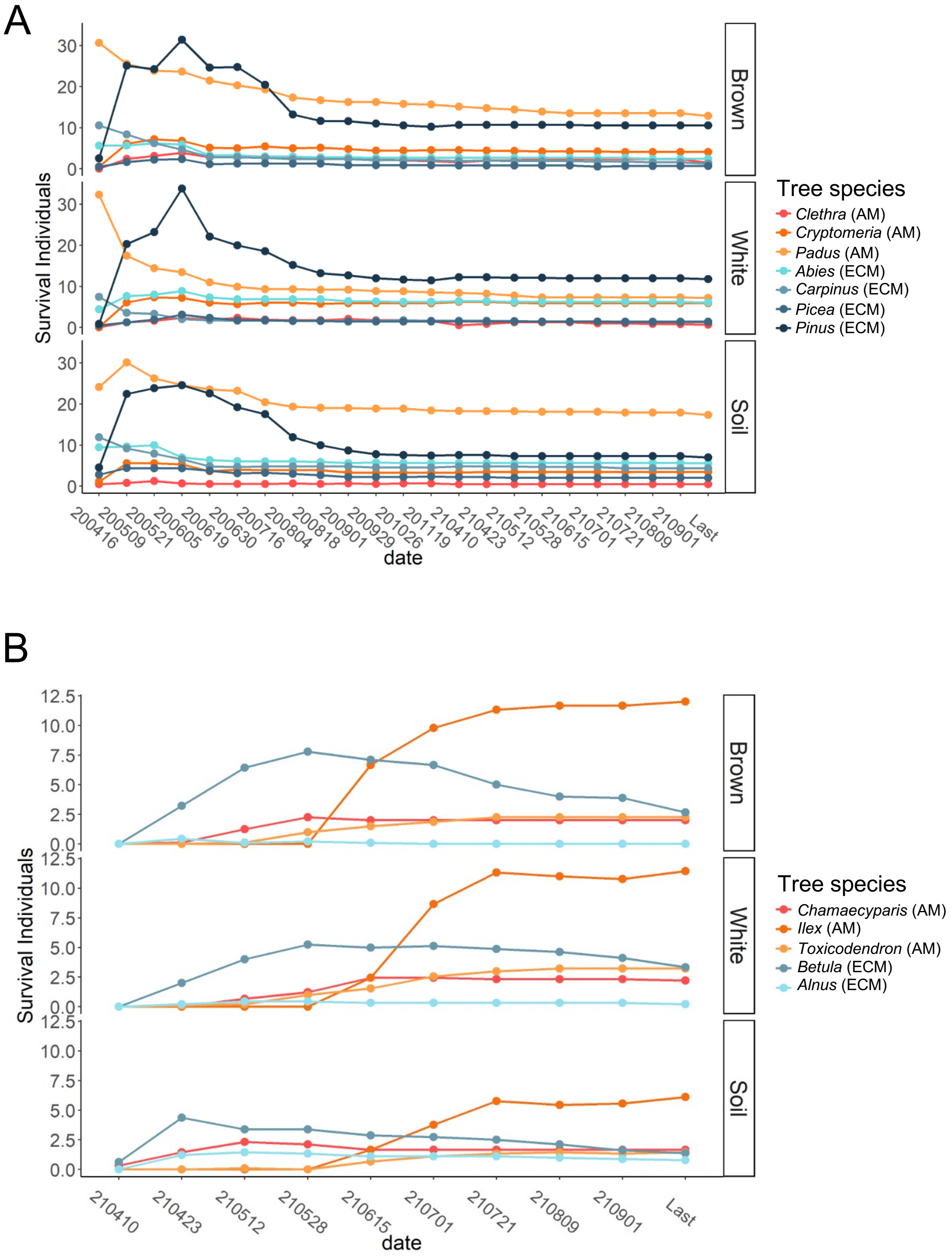

Fig. S6 Demography of germinated seedlings of trees sown in November 2019 (A) and in November 2020 (B). AM: arbuscular mycorrhizal trees. ECM: ectomycorrhizal trees. The data represents the average number of surviving seedlings in 9 quadrats. Description in the horizontal axes: Date “200416” means “16 April, 2020”; “Last” is the harvest date (October, 2021).
