# Supplementary material for: Factors associated with seedling establishment on logs of different fungal decay types—A seed‐sowing experiment
Source: Ecol Evol. 2024 Jun 4;14(6):e11508. doi: 10.1002/ece3.11508 (PMC11148398; doi:10.1002/ece3.11508)
Supplement: Supplementary file 3 — Data S1: [file ECE3-14-e11508-s005.pdf]

Factors associated with seedling establishment on logs of different fungal decay types—a seed-sowing experiment

Yu Fukasawa*, Hiroyuki Kitabatake

Laboratory of Forest Ecology, Graduate School of Agricultural Science, Tohoku University, 232-3 Yomogida, Naruko, Osaki, Miyagi 989-6711, Japan

*Corresponding author
Email: yu.fukasawa.d3@tohoku.ac.jp, Tel: +81 229 84 7397, Fax: +81 229 84 6490

Supplementary Methods

rDNA ITS sequencing

The fungal ITS1 gene region was amplified using a two-step PCR protocol with ITS1F_KYO1/ITS2_KYO2 primers (Toju et al., 2012) in primary amplification containing tails for adding indices and Illumina flow cell adapters in a secondary amplification.

1st Forward primer (ITS1F_KYO1):
CGCTCTTCCGATCTCTGNNNNNNCTHGGTCATTTAGAGGAASTAA
1st Reverse primer (ITS2_KYO2):
TGCTCTTCCGATCTGACNNNNNNTTYRCTRCGTTCTTCATC
Where the left parts of the sequence separated by repeated N are adapters to attach to the second PCR primers, and the right parts are specific sequences to the targeted ITS1 region.

Primary amplification was conducted in 10µl reaction mixture: 1.0 µl template DNA, 6.75 µl nuclease-free water, 1.0 µl 10× *Ex Taq* buffer, 0.05 µl *Ex Taq* Hot Start version (Takara Bio, Kusatsu, Japan), 0.8 µl 2.5 mM dNTP mixture, 0.2 µl forward primer (10 µM), and 0.2 µl reverse primer (10 µM). A PCR cycling protocol was conducted with an initial incubation at 94°C for 5 min, 30 cycles of denaturation at 94°C for 30 s, annealing at 50°C for 30 s, and extension at 72°C for 30 s, and a final elongation step at 72°C for 7 min.

The amplicons from the primary PCR were purified using AMPure XP (Beckman Coulter, California, USA) for removing primer dimer and size selection, and were used as template DNA for the second PCR. A second PCR reaction was set up to add the Illumina flow cell adapters and indices, using the following recipe (12 µl reaction mixture): 1.6 µl template DNA, 2.0 µl nuclease-free water, 2.4 µl *PrimeStar GXL* buffer, 0.24 µl *PrimeStar GXL Polymerase* (Takara Bio), 0.96 µl 2.5 mM dNTP mixture, 4.8 µl forward primer and reverse primer with index mix (10 µM). 2nd PCR cycling protocol was conducted with an initial incubation at 94°C for 5 min, 30 cycles of deaturation at 94°C for 30 s, annealing at 55°C for 30 s, and extension at 72°C for 30 s, and a final elongation step at 72°C for 7 min.

2nd Forward primer:

AATGATACGGCGACCACCGAGATCTACAC-index1-ACACTCTTTCCCTACACG ACGCTCTTCCGATCTCTG

2nd Reverse primer:

CAAGCAGAAGACGGCATACGAGAT-index2-GTGACTGGAGTTCAGACGTGTG CTCTTCCGATCTGAC

The concentrations of each second PCR product (libraries) were measured using a Microchip Electrophoresis System (MultiNA, Shimadzu, Kyoto, Japan) with a DNA-2500 Reagent Kit (Shimadzu). A 3 µl libraries from each sample, each with a different index, were then pooled. To reduce the salt concentration, the mixed libraries were purified twice by using AMPure XP and the buffer was replaced with elution buffer. Fragment sizes (range of 200–650 bp) in the purified library were checked using MultiNA. The final concentration was measured using a SYBR green quantitative PCR assay (Library Quantification kit; Clontech Laboratories, Mountain View, CA, USA) with primers specific to the Illumina system. The DNA library was diluted to be 8 nM by nuclease-free water, and then further diluted to 20× twice by nuclease-free water, and 10× by Easy Dilution Buffer (Library Quantification Kit; Takara Bio). Concentrations of the standard DNA in the kit were 10 pM, 1 pM, 0.1 pM and 0.01 pM. Quantitative PCR was performed in 20 µl reaction mixture: 2.0 µl diluted DNA (or 2.0 µl standard DNA), 4.0 µl nuclease-free water, 4.0 µl 5× Primer mix, 10.0 µl Terra PCR Direct SYBR Premix (Library Quantification Kit; Takara Bio). All library dilutions and standard DNA were replicated in three wells. A PCR cycling protocol was conducted with an initial incubation at 98°C for 2 min followed by 25 cycles of denaturation at

98°C for 10 s, annealing at 60°C for 15 s, and extension at 68°C for 45 s. The PCR products were stored at 4°C. Data quality was checked using the KAPA pPCR Efficiency Calculator (Kapa Biosystems), and the DNA concentration of the pooled library was calculated with a standard curve. The final products were sequenced on an Illumina MiSeq sequencer (Illumina, San Diego, CA, USA) by using MiSeq Reagent Nano Kit v2 for 2 x 250 bp PE.

References

Toju H, Tanabe AS, Yamamoto S, Sato H (2012) High-coverage ITS primers for the DNA-based identification of ascomycetes and basidiomycetes in environmental samples. Plos One 7:e40863.
